# Valuing and mapping cork and carbon across land use scenarios in a Portuguese *montado* landscape

**DOI:** 10.1371/journal.pone.0212174

**Published:** 2019-03-07

**Authors:** Marius von Essen, Inês T. do Rosário, Margarida Santos-Reis, Kimberly A. Nicholas

**Affiliations:** 1 Lund University Centre for Sustainability Studies, Lund, Sweden; 2 cE3c –Centre for Ecology, Evolution and Environmental Changes, Faculdade de Ciências, Universidade de Lisboa, Lisboa, Portugal; University of New England, AUSTRALIA

## Abstract

The ecosystem services approach can inform decision-making by accounting for both short- and long-term benefits from different land use options. Here we used the InVEST toolkit to quantify and map key ecosystem services at the largest publicly-owned agro-silvo-pastoral farmstead in Portugal–a site representative for the *montado* landscape. We analyzed how Provisioning (cork production) and Regulating & Maintenance (carbon storage and sequestration) services would be affected under three land use change scenarios, which were developed in collaboration with the forest manager of the study area: Cattle Intensification, Forest Improvement, and Residential Development. Results show that increasing cattle or residential development would deliver substantially lower levels of services. We find that extensive management, improvements to forest quality, and promotion of traditional livestock grazing would provide the highest levels of assessed ecosystem services, resulting in 13.5% more carbon storage (worth between $0.34-$7.79 million USD depending on carbon price) and 62.7% more cork production (total value of USD $3.5 million) than the current land use. However, a shift in economic incentives to make sustainable cork harvesting and traditional low-density grazing of smaller ruminants like sheep and goats profitable are likely needed to reward traditional land stewardship and help support this iconic Mediterranean landscape in the future.

## Introduction

For centuries, the *montado* cork oak savannah-like landscapes of Central and Southern Portugal have featured a biodiverse and productive mix of cork and holm oaks with crops, fallows, pastures or shrubby understory and grazing livestock. Traditional management practices have shaped an ecosystem which supports agro-silvo-pastoral production activities and provides multiple ecosystem services [1,2]. Described as low-input management system, the *montado* is traditionally used for cork and timber production, livestock grazing, sheep and goat raising, and collection of non-timber forest products such as acorns and mushrooms [2,3]. Cultural values such as nature-based tourism, traditional agricultural practices (e.g. stripping the cork by hand with a small axe) or traditional songs (the music genre *Cante Alentejano* is part of the UNESCO Intangible Cultural Heritage Lists) have been increasingly recognized for this landscape [4].

The *montado* supports high levels of biodiversity and is listed under the EU Habitats directive (habitat 6310) and represented within the Natura 2000 network [5–7]. Its heterogeneous character provides a mosaic of habitats which are home to a diverse assemblage of invertebrates and vertebrates, including endangered species such as the Iberian lynx (*Lynx pardinus*) and the Iberian imperial eagle (*Aquila adalberti*) [2,8].

The cork oak tree (*Quercus suber)* is eponymous for the cork oak *montado* and is at the center of both the local landscape and economy. Cork is a renewable resource extracted by stripping off the corky bark from the living cork oak without damaging the vital tissue layer of the tree. After the first stripping at an age of approximately 25 years, cork oaks can be commercially harvested every 9–12 years for an average period of 150 years [9]. Over the past two centuries, along with extensive sheep and goat rearing, the cork oak *montado* has been primarily managed for cork production as a result of soaring demand for wine stoppers [10].

Portugal is the world’s largest cork producer, accounting for almost 50% of global cork production [11]. The cork industry plays an important economic role at the national level, generating almost USD $990 million in exports and employing 8,295 people [11]. In the *montado*, many farms depend primarily on cork for income. Although the cork industry has undergone a diversification process which allows for the wider utilization of low-quality cork and agglomerated cork [12–14], high-quality cork bottle stoppers still play an important role in overall cork demand, making the *montado* system vulnerable to cork price volatility [15]

*Montado* landscapes are exposed to natural pressures including intense wildfires, droughts and plagues and diseases of cork oaks, as well as anthropogenic pressures related to rural land use, including both rural abandonment and the intensification of agricultural activities, particularly overgrazing by cattle [1,16]. These anthropogenic pressures are closely related to historic and ongoing socio-economic factors such as increased agricultural wages, rural migration, higher subsidies for cattle raising compared to other livestock, and volatility of cork prices [3]. Climate change exacerbates pressures like tree and forest diseases and drought, further endangering viable cork production [17].

Ecosystem services are contributions to human well-being derived from ecosystems and are grouped into the three sections Provisioning, Regulation & Maintenance, and Cultural under the CICES framework [18]. Despite the economic and ecological importance of the montado landscape, to our knowledge ecosystem service quantification remains a scarcely used support tool by land managers and spatial planers, thereby omitting an element from decision making. However, identification and quantification of ecosystem services to assess the consequences of various land use scenarios could be a central element in conserving this landscape [19]. For successful practical implementation, stakeholders should be provided with tangible and comparable information about ecosystem services for management and decision-making [20], which might include evaluations such as service quantification and monetary valuation.

In the face of a changing climate, economic uncertainty, and a dynamic EU Biodiversity Strategy, landowners need to be incentivized to maintain cork production and extensive livestock grazing regimes that support resilient landscapes and biodiversity. We explore different land use scenarios for a representative *montado* site based on past research and stakeholder input to assess how ecosystem service provision could change in a dynamic landscape under pressure. We generate a baseline for ecosystem service valuation with a focus on provisioning services (expressed through cork production) and regulating & maintenance services (expressed through carbon storage and sequestration). Specifically, we address three research questions: (1) What future land use scenarios do researchers and the land manager consider plausible? (2) How would ecosystem service delivery change for each land use scenario? (3) What is the economic value for the ecosystem services provided under each scenario? Answering these questions can inform future land use decisions in an area experiencing increasing human and environmental pressures.

## Methods

**Fig 1:**
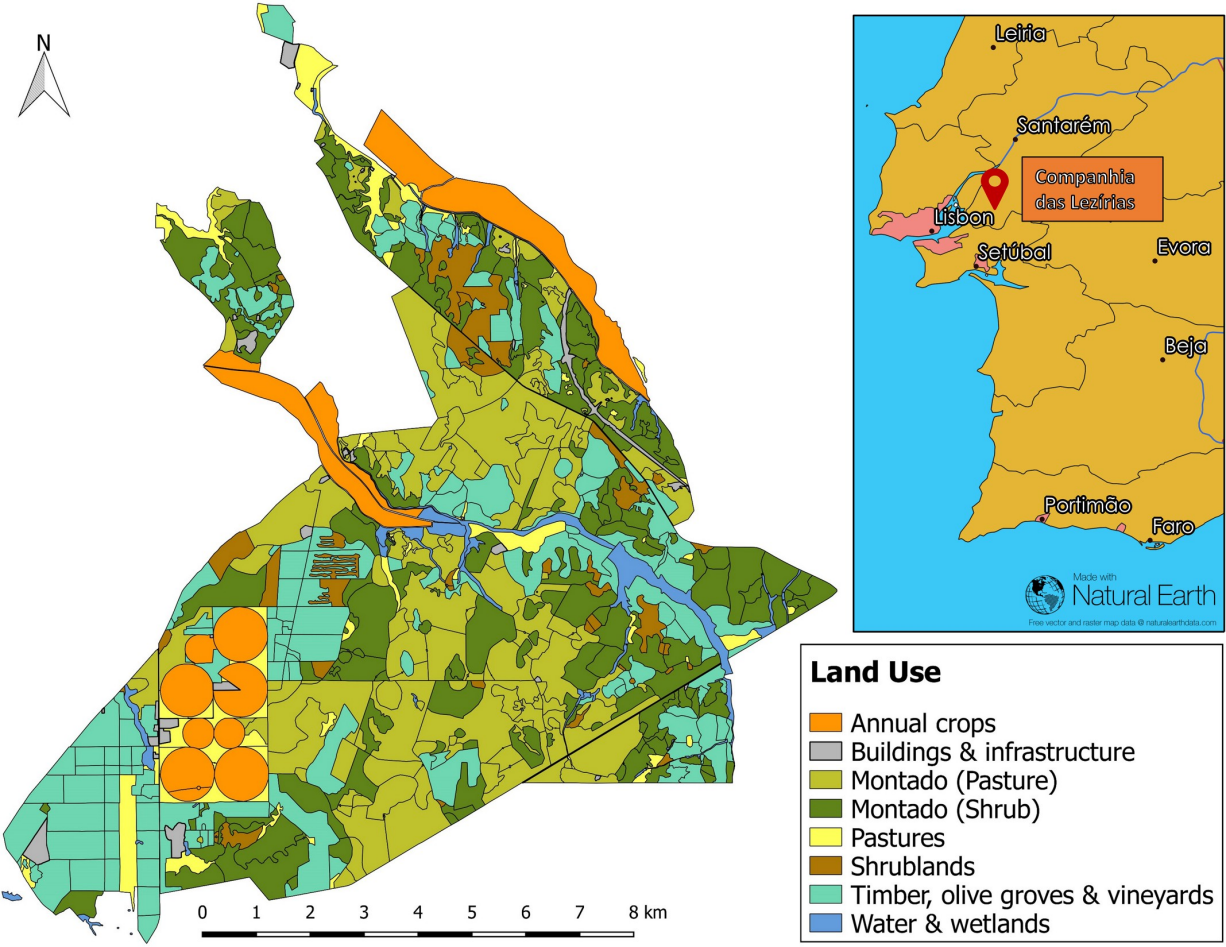
Land use distribution map of the study area. Current land use composition of the study site at the farmstead Companhia das Lezírias S.A., Portugal, about 50 km outside Lisbon. Adapted from Gonçalves, Alcobia, Simões, & Santos-Reis [26] and last updated in 2013 by Curveira-Santos, Marques, Björklund, & Santos-Reis [27], inset map: Openstreetmap showing Portugal (grey border) and location of the farmstead (pin).

Our study was based at Companhia das Lezírias S.A., the largest agro-silvo-pastoral farmstead in the Alentejo region [21,22]. Established in 1836, Companhia das Lezírias (*Companhia*) has a strong historical legacy and is operated as a public limited company wholly-owned by public funds since 1989 [21]. *Companhia* follows a ‘philosophy of a sustained development’ and aims to ‘preserve, value and make use of its patrimony and resources through an integrated, sustainable management that contributes to the needs of society and the agroforestry sector’ [21]. The farmstead is located 50 km north-east of the capital Lisbon and comprises around 18,000 hectares, 5,814 ha of which are covered with cork oak stands (*montado*) with varying understory vegetation including permanent and natural pastures as well as dense and sparse shrubs (Fig 1). Currently the farm owns around 3,000 cattle heads, which graze on open or *montado* pastures in herds of 50–300 animals. The farmstead’s cork oaks are protected by Portuguese law, which protects this species from cutting [23,24]. Further, parts of *Companhia* are protected from conversion through the Natura 2000 network and the *montado’s* only payment for ecosystem services scheme, the privately-funded Green Heart of Cork project, organized by WWF in collaboration with the Forest Producers Association of Coruche (APFC) to reward the positive influence of Forest Stewardship Council (FSC) certified *montado* for the conservation of watersheds in the targeted areas [25].

We analyzed the current land use map as well as the three land use scenarios using the spatially explicit InVEST toolkit (https://www.naturalcapitalproject.org/invest/) to map, quantify and compare ecosystem services provision [28,29]. We employed two InVEST models: the ‘Managed Timber Production’ model to quantify cork provisioning, and the ‘Carbon Storage and Sequestration: Climate Regulation’ model to quantify carbon. InVEST generates bio-physical as well as monetary results when including a user-defined valuation for the ecosystem services under consideration–in our case cork and carbon sequestration.

The land use maps used in both InVEST models were adapted from previous maps of the study site generated by Gonçalves, Alcobia, Simões, & Santos-Reis [26] and last updated in 2013 by Curveira-Santos, Marques, Björklund, & Santos-Reis [27]. In addition to quantification and valuation of ecosystem services, both models produce vector maps. The Carbon model uses five carbon pools to calculate storage and sequestration: above-ground biomass [30–33], below-ground biomass [32,34–36], soil carbon [37,38], dead organic matter [33,39], and harvested wood products (calculation based on annual reports from *Companhia*, Companhia das Lezírias, Relatórios 2011–2015). The model assumes that carbon storage within each land use type is constant over time, thus accounting only for changes in harvested wood products, not natural variation, if the land use type remains unchanged [29].

The timber model uses a set of harvest information including harvest frequency, harvested biomass, and market value of harvested products to quantify harvested mass, volume and economic value of the timber product–in our case cork. It allows for monetary valuation by subtracting maintenance and harvest costs from the market price of cork. The model assumes that certain values remain constant over the defined time period, such as harvest frequency, cost, yield, and market price [29]. We chose to express values in terms of the 50-year valuation because of the slow growth rate of cork oaks and the associated period before cork oak can be commercially harvested.

Monetary valuation of ecosystem services requires putting a price on biophysical production. To determine the revenues from cork sales, we identified the market price of $1.33 USD/kg cork that *Companhia* generated over the past 5 years (Companhia das Lezírias, Relatórios 2011–2015) at an average exchange rate of EUR 1 to USD 1.107. For the Carbon Sequestration model we employed a 7% discount rate over a period of 50 years [29].

Carbon markets are volatile and prices between carbon standards vary greatly. As of today, there is no single, globally representative price for carbon and current market values for carbon remain significantly lower compared to recommended prices deemed adequate for maintaining temperature change below 2°C [40]. To represent the current market situation and incorporate future price recommendations, we used prices of three different carbon standards: High-Level Commission on Carbon Prices 2017 ($75 USD/tCO_2_eq), IPCC AFOLU low utilization ($20 USD/tCO_2_eq) [41], and the average price of voluntary carbon market standards in 2015 ($3.3 USD/tCO_2_eq) [42].

## Scenarios

To evaluate ecosystem services from cork production and carbon sequestration under different land uses, we developed three scenarios representing plausible future development trajectories for the farmstead, resulting in new land use maps for analysis. To develop the scenarios, we drew on the results of previous research collaborations between the Science Faculty of Lisbon University and the farmstead [26], assumptions regarding plausible land use composition and consulted with the farmstead’s forest manager to ensure the plausibility of the scenarios. We defined two scenarios representing an investment into one of the two main activities at *Companhia* (cork and cattle respectively), as well as a third scenario where agroforestry revenue is assumed to decrease and part of the land is sold and/or used for scattered, low density housing development. We decided collaboratively in a meeting and through prior communication with the research team and the land manager which land uses should be converted (e.g. *montado* to pasture), the degree of change for landscape attributes (e.g. tree density) and where these changes would take place. The residential development scenario took into consideration that 8% of *Companhia’s* territory is a Nature Reserve and 64% is included in the *Special Protection Area* and Site of *Community Importance* of the Tagus estuary, thus cannot be developed. Of the remaining unprotected land, we allocated 10 ha for residential development.

**Fig 2:**
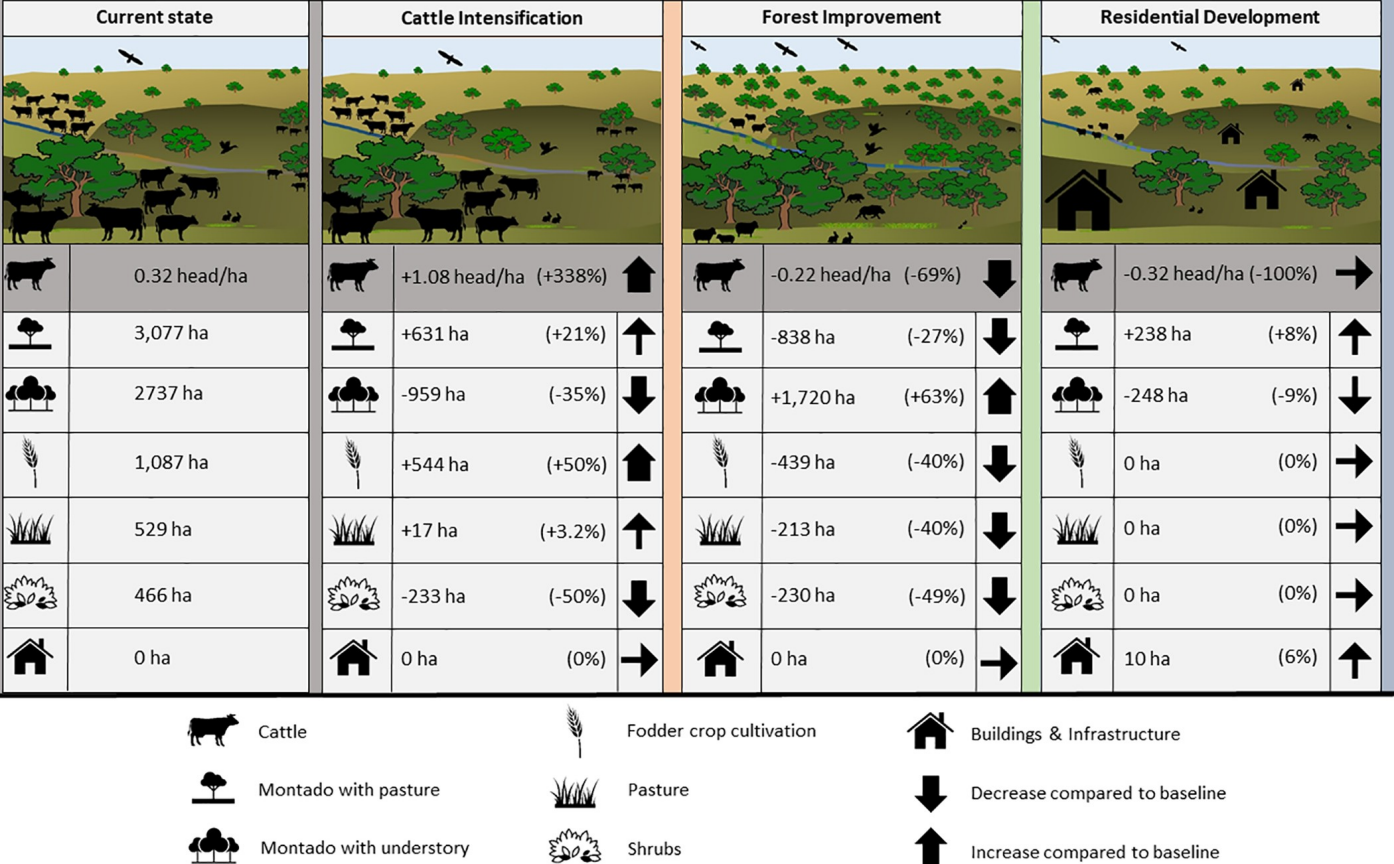
Land use distribution in current state and scenarios. Visual representations of the three future land use scenarios developed by consulting the forest manager of Companhia das Lezírias. Numbers shown are absolute for number of cattle per hectare and percent change compared to current area of each land use.

The first scenario, Cattle Intensification, entails more than tripling cattle density compared to the current density, from 0.32 under the current land use to 1.4 heads ha^-1^ –the upper limit of extensive livestock farming as defined by the EU Common Agricultural Policy [43,44]. This would require an increase in the area for fodder crop cultivations (used as a supplementary feed) and more grazing land (*montado* with pasture) to support a larger number of cattle. Higher grazing pressure from the increase in cattle reduces the area of *montado* with understory vegetation (see Fig 2).

The second scenario, Forest Improvement, considers an investment in cork production, expressed by a 50% increase in cork oak density from 80 to 120 trees ha^-1^. Focusing on cork production corresponds with a reduction of the number of cattle, since cork sales would be the major income for the farmstead and high numbers of cattle impede the expansion of cork oak *montado* as the animals feed on shoots and compact the soil. Because of the decreased number of cattle, less fodder crop cultivation area and pastures are needed to support cattle and are planted instead with cork trees. Shrub lands would also be partially replaced by *montado* with cork oaks (Fig 2). This scenario would be more probable in the case of reduced profits from cattle rearing or increasing demand for cork, which could trigger an expansion of the *montado* within the farmstead with a focus on cork production (see Fig 2), in line with *Companhia’s* sustainable management philosophy.

Alternatively, in the third scenario parts of *Companhia’s* land would be sold and/or used for housing development (Residential Development), concentrated in two areas of 602 ha total (3.3% of the study area). To maintain a visually appealing landscape which features the characteristics typically associated with cork oak *montado*, shrubby understory vegetation would be replaced by pasture alongside a reduction of tree density to 20 trees ha^-1^ (Fig 2). While cork oak trees themselves are protected by law (DL n°169/2001 de 25 de Maio), exceptions can be made in cases of national interest. The region's proximity to Lisbon has made it an attractive location for both exurban and recreational development resulting in. Similar development projects have been realized nearby such as the Orizonte Lisbon Gold [45] and Montado Hotel & Golf Resort [46]. The extension and conversion of the Montijo Air Base into a commercial airport for Lisbon would further increase the pressure to develop this *montado* for infrastructure, rural tourism, or residential projects [47]. However, according to *Companhia’s* land manager large scale housing development in the study area is unlikely to happen rendering the Residential Development scenario the least likely.

**Fig 3:**
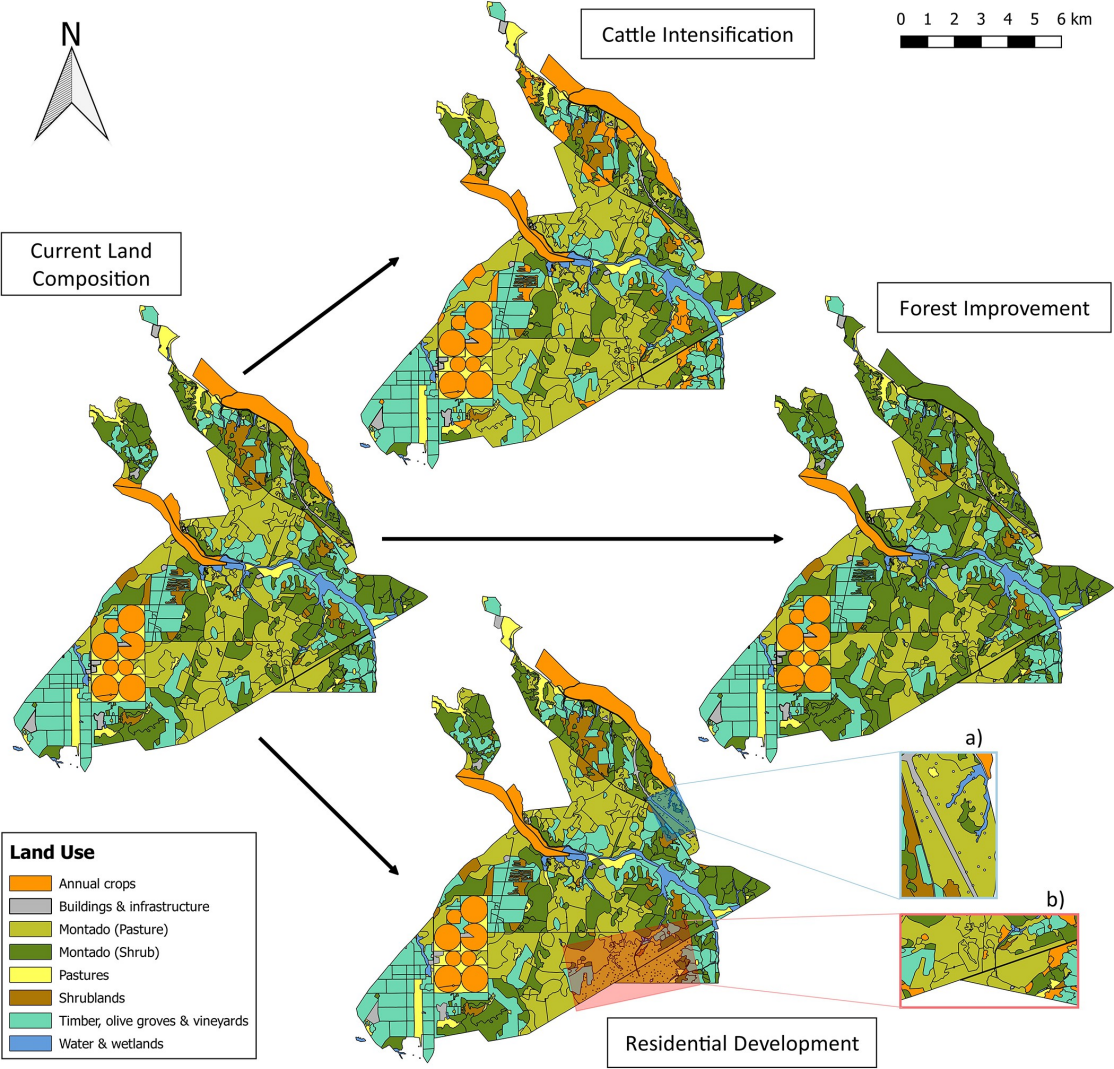
Land use composition maps for current state and scenarios. Study area maps showing the baseline scenario (current land use composition) and how land use would be changed under the three future land use scenarios described in Fig 2. Inset map a) shows the North-West residential development area, inset map b) the South-West residential development area.

The most significant land use changes under the three future scenarios take place in the central and eastern parts of the study site (Fig 3), representing *montado* with pasture and *montado* with shrubs along with areas for fodder crop cultivation. The south-western tip of the study area remains mostly unchanged, retaining its current configuration of olive groves, vineyards, and areas for fodder crop cultivation, and the water bodies and riparian areas are also undisturbed in the future scenarios (Fig 3).

## Results

The Forest Improvement scenario yielded the highest levels of both carbon storage and cork production, while the Residential Development scenario delivered close to current provision levels, and Cattle Intensification scores lowest. Forest Improvement stores 13.5% more carbon annually compared to the current land composition, resulting in 104 Gg of sequestered carbon over a 50-year period (Table 1). Carbon storage under the Residential Development scenario decreased by 29 Gg (-3.8%) and by 83 Gg (-11%) under Cattle Intensification.

**Table 1:** Cork biomass production and carbon sequestration in Gg over a 50-year period.

	Current Land Composition	Cattle Intensification	Forest Improvement	Residential Development
Total cork biomass production (Gg)	6.5	5.5	10.5	6.0
Revenue from cork sales (million USD)	14.32	$23.31	$12.20	$13.23
Carbon sequestration (Gg)	-	-82.6	103.9	-28.9

In the Forest Improvement scenario cork production increases from 6.5 Gg to 10.5 Gg of biomass (+61.5%) while in the Cattle Intensification scenario production declines by 1.0 Gg compared to the current land composition (-15.4%). Cork production under Residential Development drops to 6.0 Gg (-7.7%).

Converting the production of our studied ecosystem services into monetary values corroborates the trend observed for bio-physical values: Forest Improvement generates the highest amount of potential revenue from ecosystem services while Cattle Improvement produces the lowest.

Based on the 5-year average market price of *Companhia’s* cork products, we calculated revenue from cork sales of $23.31 million USD for the Forest Improvement scenario. In contrast, the revenue from cork sales in the Cattle Intensification scenario is lower by $11.11 million USD (-47.7%), totaling $12.20 million USD (Table 1) while the Residential Development scenario generates $13.23 million USD of sales revenue.

**Fig 4:**
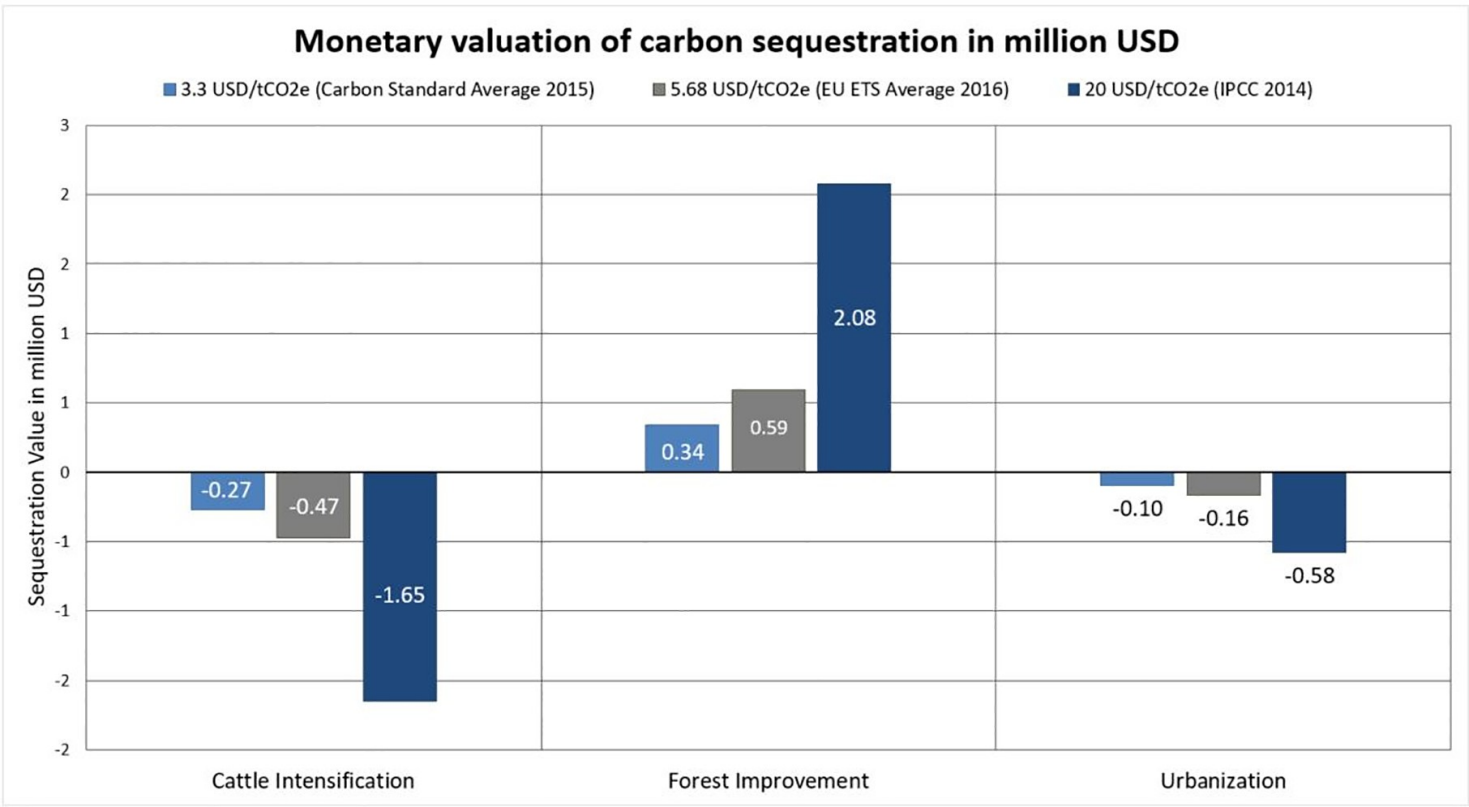
Carbon sequestration value for a 50-year period in million USD. Carbon sequestration value calculated using the three sources for prices shown, based on the carbon sequestration calculated by InVEST for the three land use scenarios (results shown in Table 1).

The monetary valuation of carbon sequestration provides a similar picture. Forest Improvement shows positive income from sequestration, with values ranging from $0.34 to $7.79 million USD over 50 years while Cattle Intensification (-$0.27 to -$6.20 million USD) and Residential Development (-$0.10 to -$2.27 million USD) represent losses (Fig 4).

Although the trends for carbon sequestration are the same between scenarios, varying the value for the price of carbon results in stark differences of absolute monetary value between the scenarios. When applying the average price of voluntary carbon market standards in 2015 ($3.3 USD/tCO_2_eq) [42], the absolute difference in sequestration value between Cattle Intensification and Forest Improvement is $0.61 million USD. The same two scenarios, however, show an absolute margin in sequestration values of $13.99 million USD when applying the IPCC 2014 AFOLU value for carbon–more than a six-fold difference.

When combining the monetary values of both carbon storage and cork production, differences in ecosystem services values between the scenarios increase further: the Cattle Intensification scenario generates a total ecosystem service value between $-0.5 million to $5.23 million USD. The total value of carbon sequestration and cork provision in the Forest Improvement scenario ranges from $10.84 million to $18.29 million USD and in the Residential Development from $3.83 million to $5.9 million USD.

## Discussion

The *montado* landscape is subject to both anthropogenic (e.g. agricultural intensification, fluctuating demand for cork, rural abandonment) and natural pressures (e.g. drought, wildfires), challenging traditional cork production practices and extensive livestock grazing regimes which are vital for maintaining the *montado’s* ecological resilience and biodiversity [10,15]. However, agricultural policies and subsidy schemes, dominant land management philosophies, and inadequate carbon pricing mechanisms instead favor intensive cattle production over ruminants traditionally found in the *montado*, such as sheep and goats [48,49]. In addition, revenue from cork production is subject to market demands and price volatility, despite a diversification process of the cork industry [12–15]. Climate change further exacerbates this issue with a temperature increase of 2.9°C estimated to decrease cork production by 20% [50]. Thus, including alternative indicators like ecosystem service provision into decision and policy making processes may generate additional incentives for sustainable use in the *montado* [51]. A frequently suggested solution for integrating ecosystem services into decision making processes and incentivizing sustainable practices is payment for ecosystem services (PES) [52]. With the Green Heart of Cork project, a PES scheme has been established in the Portuguese Alentejo and Ribatejo regions in which *Companhia* also partakes [25]. The scheme recognizes the positive influence of High Conservation Value area and Forest Stewardship Council (FSC) certified *montado* for the conservation of watersheds [2,15,53]. Participating land owners receive $17.80 USD per hectare of high conservation value area, totaling $11,070 USD per annum for *Companhia* [54,55]. Further, the farmstead participates in the *Carbono Zero* project, through which they sold 1,300 t CO_2_ from 55ha of *montado* in 2017 [56].

These two examples of comparatively low and/or infrequent income generated through PES schemes point towards a critical issue: valuation of ecosystem services does not directly translate into income for the participants. While in the case of *Companhia* cork is a direct source of income, carbon sequestration and watershed protection generate negligible profits. For carbon, this is mostly due to the absence of a viable carbon sequestration payment system, high market volatility, and low average prices [42].

In addition to cork production as a provisioning service and carbon sequestration as a regulation and maintenance service, we attempted to utilize the ‘Visitation: Recreation and Tourism’ model for cultural valuation, but the online database Flickr, used by the InVEST model, had too few entries of geotagged photographs to generate significant results. However, related work has shown that traditional *montado* was the most valued landscape by participants of a Portuguese study carried out in 2016 [57]. The high aesthetical and cultural appreciation of the *montado* could provide a further pathway for landowner to capitalize on maintaining and adopting sustainable management practices. Future studies will benefit from systematically selecting ecosystem services to represent the three CICES sections of ecosystem services (Provisioning, Regulation & Maintenance, and Cultural) in order to capture potential tradeoffs [18].

A further opportunity to highlight the value of a healthy *montado* lies in its capacity to contribute to carbon mitigation targets. With 5,814 ha of cork oak woodland, *Companhia* accounts for 0.79% of Portugal’s total *montado* landscape (737,000 ha) [58]. Under the Forest Improvement scenario, this area would sequester 2,000 tons of carbon annually, enough to offset the annual carbon emission of more than 1,300 passenger cars (calculated at an average annual kilometrage of 14,000 km and average emissions of 106 g CO2/km) [59]. Given the extensive areas of *montado* in Portugal, an increase in tree density along with a forest-friendly management style provides an opportunity to significantly contribute to Portugal’s emissions reduction targets. If the total area of *montado* would be restored to 1995 levels, accompanied by a higher tree density like the Forest Improvement Scenario, an additional 11,000 tons of CO_2_ could be sequestered annually.

The Forest Improvement scenario describes an ambitious management trajectory that would require commitment from landowners. Our collaboration with *Companhia* indicates they may be willing to undertake this commitment; if they did so, they might function as an exemplar for the implementation of forest-friendly management in *montado* landscapes.

## Conclusion

The *montado* is a beautiful, traditional cultural landscape now facing many pressures. The choices that land managers make will shape one of many possible futures for the *montado*; here we explored three possible trajectories for this future in conjunction with a forest manager of a large farmstead. We found that both cork production and carbon storage were increased under a Forest Improvement scenario that favored traditional management. However, current economic incentives favor maximized cattle production and disincentive extensive cork production, to the detriment of the biodiversity, cultural heritage, and potentially long-term productivity of the *montado*. Finding new economic incentives to make sustainable cork harvesting and traditional low-density grazing of smaller ruminants like goats profitable, whether through a reformed Common Agricultural Policy, a stronger carbon market, or otherwise, are likely needed to reward traditional land stewardship and help support this iconic Mediterranean landscape thriving into the future.
